# Linking plant and vertebrate species to Nature’s Contributions to People in the Swiss Alps

**DOI:** 10.1038/s41598-023-34236-2

**Published:** 2023-05-05

**Authors:** Pierre-Louis Rey, Pascal Vittoz, Blaise Petitpierre, Antoine Adde, Antoine Guisan

**Affiliations:** 1 Institute of Earth Surface Dynamics, Faculty of Geosciences and Environment, University of Lausanne, Lausanne, Switzerland; 2 Info Flora, c/o Conservatoire et Jardin botaniques de Genève, Chambésy-Genève, Switzerland; 3 Department of Ecology and Evolution, Faculty of Biology and Medicine, University of Lausanne, Lausanne, Switzerland

**Keywords:** Biodiversity, Ecosystem services, Ecology, Conservation biology

## Abstract

Since the late 1990s, Nature’s Contributions to People (NCPs; i.e. ecosystem services) were used as a putative leverage for fostering nature preservation. NCPs have largely been defined and mapped at the landscape level using land use and cover classifications. However, NCP mapping attempts based directly on individual species are still uncommon. Given that species shape ecosystems and ultimately deliver NCPs, mapping NCPs based on species distribution data should deliver highly meaningful results. This requires first establishing a census of the species-to-NCP relationships. However, datasets quantifying these relationships across several species and NCPs are rare. Here, we fill this gap by compiling literature and expert knowledge to establish the relationships of 1816 tracheophyte and 250 vertebrate species with 17 NCPs in the Swiss Alps. We illustrated the 31,098 identified species-NCP relationships for the two lineages and discuss why such a table is a key initial step in building spatial predictions of NCPs directly from species data, e.g. to ultimately complement spatial conservation planning.

## Introduction

Despite decades of attempts to improve nature conservation^1^, the degradation of biodiversity has continued and even worsened worldwide^1–3^, and awareness of humans for the value of nature and the reasons to preserve it was still insufficient at the beginning of the second millenary^4,5^. As an attempt to improve this, the concept of ‘Ecosystem Service’ (ES), recently reframed in the larger concept of ‘Nature’s Contributions to People’ (NCPs) by the Intergovernmental Science-Policy Platform on Biodiversity and Ecosystem Services^6^ (IPBES), was developed with the aim of ‘bending the curve’ of biodiversity loss^7^ by highlighting its potential values for the socio-economic development and well-being of humans^8^. Although NCPs and ES are not fully interchangeable terms^9^, for sake of clarity we adopt here the terminology of the common international classification of ES (CICES^10,11^) as basis to select the NCPs for our study. However, whereas protected areas were established worldwide to preserve endangered species or habitats^12,13^, they were not necessarily designed to preserve associated ecosystem services and cultural values, despite being included in the definition of protected areas proposed by the IUCN^12,14^. There is thus a need to map NCPs spatially, e.g. to include them in spatial conservation prioritization^15^.

Large efforts were made to map NCPs in geographic space^16^ and to include them in spatial conservation planning^17^. Yet, the mapping of NCP was often performed at the level of coarse landscape units^18^ (e.g. ecosystems, habitat units, etc.), which tend to blur the complexity of the relations between species and NCPs^19^ (e.g. the use of conifers and decideous trees layers (no distinction between species) to characterize timber production^17^). Moreover, most studies combining species and NCPs were either comparing or opposing them as separate features^20^. However, global changes and their impact on species distributions^21^ will reshuffle the species composition of current land units, potentially affecting the NCPs associated with them. Therefore, properly assessing the future capacity of landscape units and their ecosystems to deliver NCPs likely also requires direct association of NCPs with individual species.

Yet, there is currently a critical lack of information on the linkages between NCPs and species^18,22,23^ across large number of species and NCPs. As our capacity to predict NCP in time and space is still limited^19^, building a species-NCP relationship table would allow predictions of NCPs at sites where information on the presence or abundance of multiple species is available, be it real observations or predictions. As healthy ecosystems are built on complex networks of species interactions^24^, it is important to include as many species as those potentially contributing to ecosystem functioning or service delivery, and to consider a set of NCPs that can be predicted from the set of selected species, also potentially considering their specific traits^25^.

Thus far, most of the species-NCP studies have focused on vascular plants^18,22,26,27^. Among existing attempts, a first study crossed 171 tree species with 15 Japanese culture-related NCPs^26^. Recently, a study compiled a dataset of plant use records for all accepted vascular plant genus (13,489 genera)^27^, based on the most comprehensive global review of plant classification and their uses^28^. The latter produced a review of the functional aspects of each vascular plant (only direct uses), and without consideration of any potential negative relationships to NCPs. Finally, conservation planning studies that simultaneously consider species distributions, NCPs, and protected areas are still extremely rare, and existing attempts have shown that current protected areas may not match future species and NCP needs^21,29,30^. Furthermore, considering species or NCPs separately may lead to conflicting outcomes^17,20,31^.

To help ecological conservation studies move a step forward, we evaluated the relationships between 2066 terrestrial tracheophyte and vertebrate species and 17 NCPs in part of the Swiss Alps, by creating a two-way contingency table compiling their positive, neutral, and negative relationships, as identified from the literature and expert knowledge. We focused on these two lineages because the largest information on their roles and functions exists and could be used to document their relationships with NCPs. They are also the taxa most investigated by researchers^32^, most often used in conservation analyses^33,34^, and most popular to the public^32^. We illustrated the potential of the relationship table for predicting NCPs from species observations and discussed further applications to derive spatial NCP predictions from individual species predictions.

## Results

**Figure 1:**
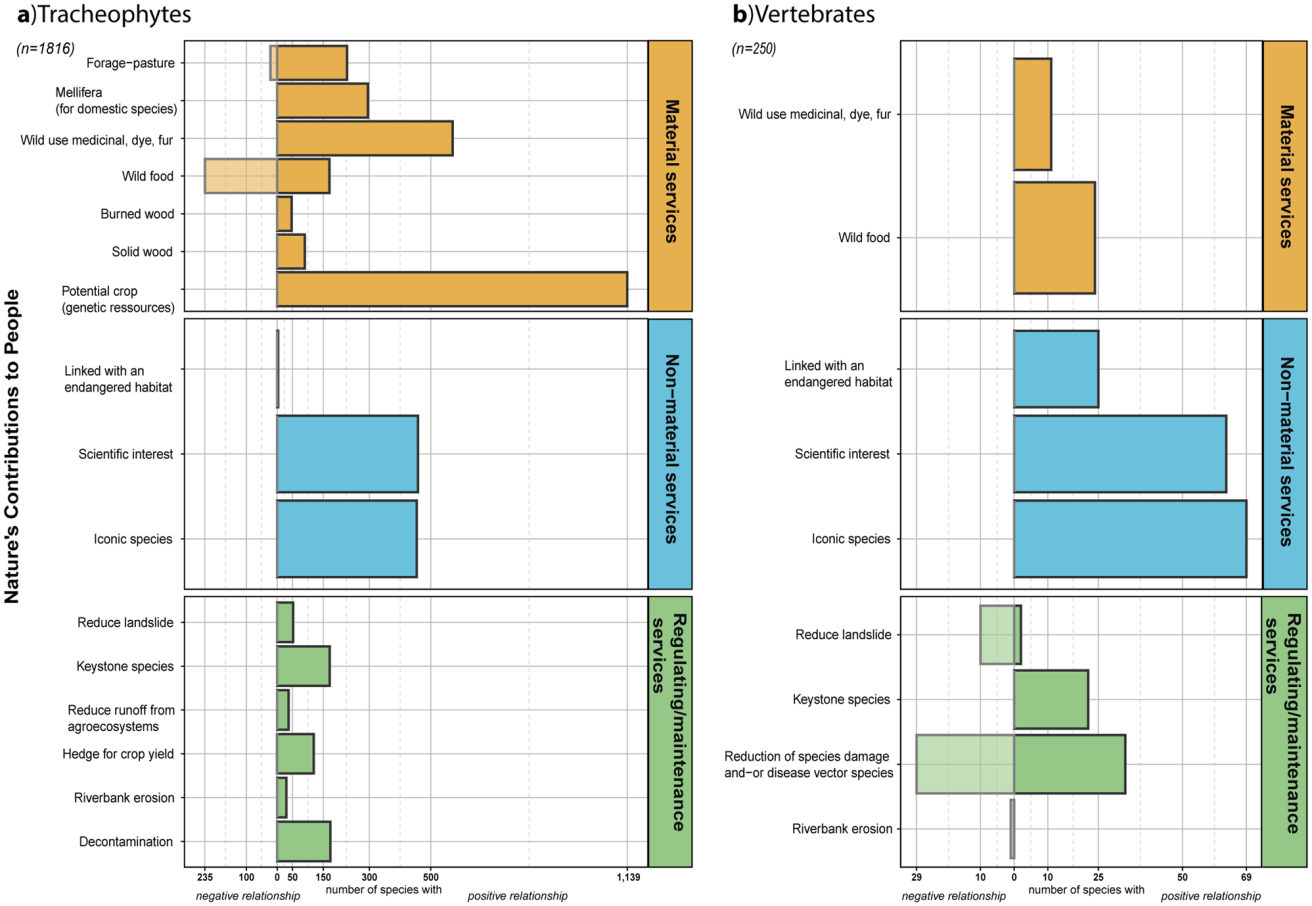
Number of reported relationships between the (**a**) 1816 tracheophyte and (**b**) 250 vertebrate species with 17 target NCPs (8 common to both groups, 8 and 1 restricted to tracheophytes and to vertebrates, respectively). Dark bar illustrates the number of species with a positive relationship to each NCP. Light bar illustrates the number of species with a negative relationship to each NCP. The number of neutral species corresponds the difference between the total number of species and the species with positive or negative relationships.

We reported 31,098 individual relationships (out of 31,306 possible) linking the target 17 NCPs to the 2066 species. The total resulting species-NCP relationship table is available on the Appendix S3 (see ‘Data availability’) and Fig. 1 displays a graphical summary. The NCP with the highest number of species for a positive relationship concerned only tracheophyte species and it was “potential crop” with 1139 (62.7%) of the 1816 species. The NCP with the highest number of species expressing a negative relation was “wild food” with 235 (12.9%) species (also established with tracheophytes, Fig. 1a).

### Tracheophyte species

**Figure 2:**
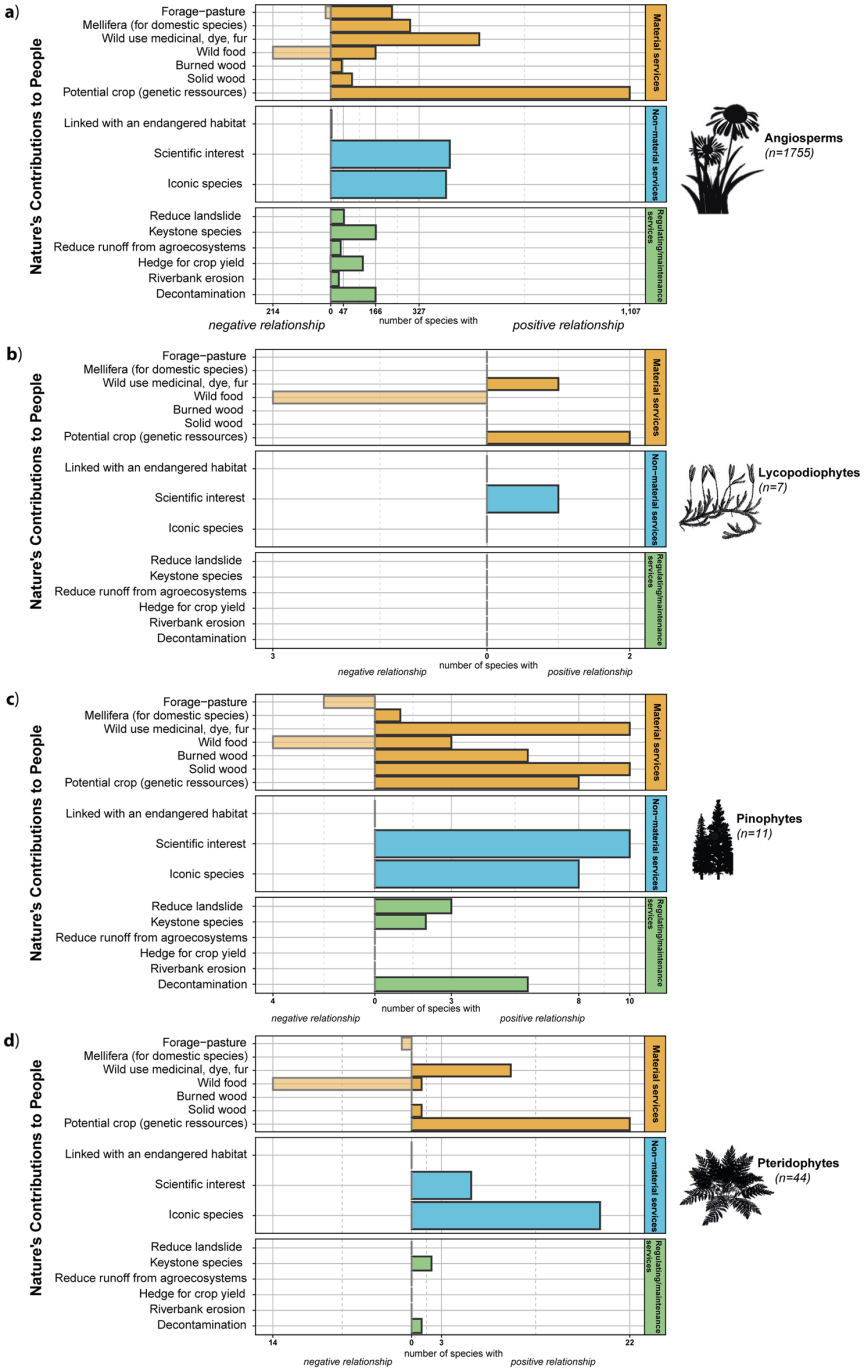
Number of reported relationships between the 1816 tracheophytes species and 16 target NCPs. Dark bar illustrates the number of species with a positive relationship to each NCP. Light bar illustrates the number of species with a negative relationship to each NCP. The number of neutral species corresponds to the difference between the total number of species and the species with positive or negative relationships.

Of the 1816 tracheophyte species, 1139 (62.7%) had a positive link with the “potential crop” NCP. Tracheophyte species were very often positively linked to non-material NCPs, in particular with 458 species (25.2%) for the “scientific research interest” NCP and 454 (25%) for the “iconic species”. Only three species were linked to the “endangered habitats” NCP. Among tracheophytes, the highest number (i.e. best documented) of species-NCP relationships was for the angiosperm group (Fig. 2), with up to 97.2% of the “potential crop” NCP covered by this class. The pinophyte group (Fig. 2c) exhibited positive relationships with all except one species (10 out of 11, i.e. 90.9%). There was paucity of information concerning the relations of lycopodiophyte species with NCPs (Fig. 2b). The same observation was made for the pteridophyte group (Fig. 2d) with the regulating/maintenance NCP category.

### Terrestrial vertebrate species

**Figure 3:**
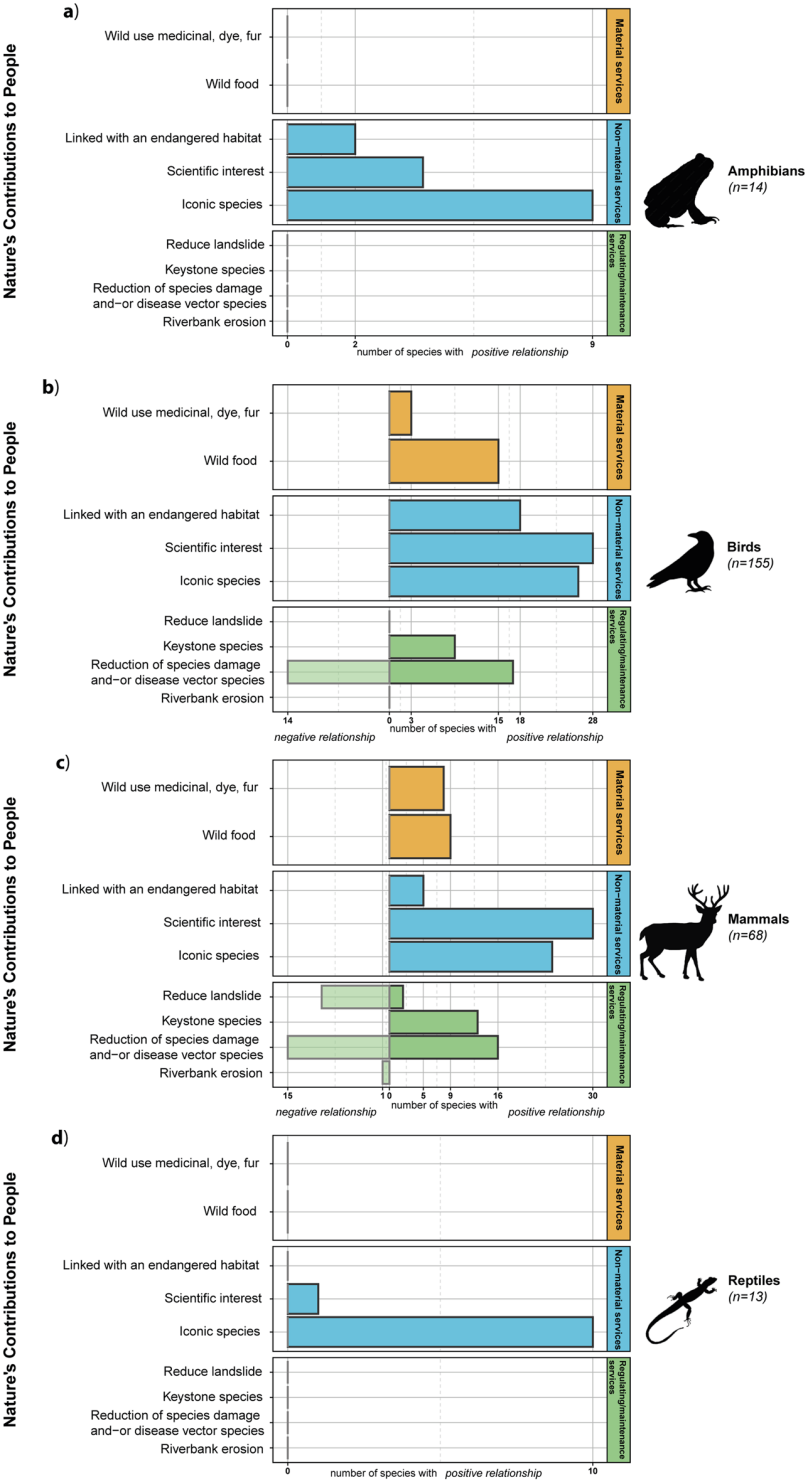
Number of reported relationships between the 250 vertebrate species and 9 NCPs. Dark bar illustrates the number of species with a positive relationship to each NCP. Light bar illustrates the number of species with a negative relationship to each NCP. The number of neutral species corresponds to the difference between the total number of species and the species with positive or negative relationships.

With 250 species concerned, the highest number of positive relationships between NCPs and terrestrial vertebrates was only 69 (27.6%) and all were associated with “iconic species”. We found only positive relationships between vertebrates and both material and non-material NCPs. Results were different for NCPs from the regulating/maintenance NCP category, showing 34 (13.6%) positive and 29 (11.6%) negative relationships with the “reduction of species damage and disease vector species” NCP. This was also the case for the “reduce landslide” NCP, with 2 and 10 species exhibiting positive and negative relationships, respectively. In the same regulating/maintenance NCP category, the “keystone species” NCP had only a positive relationship with species (n = 22; 8.8%). Among the vertebrate groups, the number of relationships (positive and negative) was roughly proportional to the species richness of each subgroup: 130 relationships for birds, 133 for mammals (Fig. 3b,c), 15 for amphibians, and 11 for reptiles, and were only linked to non-material services (Fig. 3a,d).

Table 1 displays a sample of the raw content of the relationship table for the five species with the highest NCP score (i.e. the number of reported relationships between species and NCPs). The sub-table for tracheophyte species (Table 1A) shows that frequent, pioneer, and alluvial species have the highest NCP score, and willow species (*Salix alba*, *Salix caprea*) have a 75% positive relationship with NCPs. The sub-table for vertebrate species (Table 1B) is more contrasted, with the greatest number of positive relationships obtained for three predators and two emblematic birds from high-elevation areas.

**Table 1:** Sample of the species-NCP relationship table for the five tracheophytes (a) and vertebrates (b) species with the highest number of reported positive relationships with NCPs.

(a) Tracheophytes	Material services										Non-material services						Regulating/maintenance services											
Scientific name English name	Potential crop*	Solid wood	Burned wood	Wild food	Wild use*	Mellifera*	Forage-pasture	TOTAL MAT n = 7	TOTAL MAT positive	TOTAL MAT negative	Iconic species	Scientific interest	Linked to*	TOTAL NON-MAT n = 3	TOTAL NON-MAT positive	TOTAL NON-MAT negative	Dec*	Riverbank erosion	Hedge for crop yield	Reduce runoff *	Keystone species	Reduce landslide	TOTAL REG n = 6	TOTAL REG positive	TOTAL REG negative	TOTAL NCPs n = 16	TOTAL NCPs positive	TOTAL NCPs negative
***Salix alba*** White willow	1	1	1	0	1	1	1	**6**	6	0	1	1	0	**2**	2	0	1	1	0	0	1	1	**4**	4	0	**12**	12	0
***Salix caprea*** Goat willow	1	1	0	0	1	1	1	**5**	5	0	1	1	0	**2**	2	0	1	0	1	1	1	1	**5**	5	0	**12**	12	0
***Populus tremula*** Aspen	1	1	1	0	1	1	0	**5**	5	0	1	1	0	**2**	2	0	1	1	1	1	0	0	**4**	4	0	**11**	11	0
***Alnus glutinosa*** Common alder	1	1	1	0	1	1	0	**5**	5	0	1	1	0	**2**	2	0	0	1	1	0	0	1	**3**	3	0	**10**	10	0
***Betula pendula*** White birch	1	1	1	0	1	0	1	**5**	5	0	1	1	0	**2**	2	0	1	0	1	1	0	0	**3**	3	0	**10**	10	0

(b) Vertebrates	Material services					Non-material services						Regulating/maintenance services																
Scientific name English name	Wild food	Wild use*	TOTAL MAT n = 2	TOTAL MAT positive	TOTAL MAT negative	Iconic species	Scientific interest	Linked to*	TOTAL NON-MAT n = 3	TOTAL NON-MAT positive	TOTAL NON-MAT negative	Riverbank erosion	Reduction of species damage*	Keystone species	Reduce landslide	TOTAL REG n = 4	TOTAL REG positive	TOTAL REG negative	TOTAL NCPs n = 9	TOTAL NCPs positive	TOTAL NCPs negative							
***Vulpes* vulpes** Red fox	0	1	**1**	1	0	1	1	0	**2**	2	0	0	1	1	0	**2**	2	0	**5**	5	0							
***Lynx lynx*** Eurasian lynx	0	0	**0**	0	0	1	1	1	**3**	3	0	0	0	1	1	**2**	2	0	**5**	5	0							
***Lagopus muta*** Rock ptarmigan	1	1	**2**	2	0	1	0	1	**2**	2	0	0	0	0	NA	**0**	0	0	**4**	4	0							
***Lyrurus tetrix*** Black grouse	1	1	**2**	2	0	1	0	1	**2**	2	0	0	0	0	NA	**0**	0	0	**4**	4	0							
***Canis lupus*** Grey wolf	0	0	**0**	0	0	1	1	0	**2**	2	0	0	-1	1	1	**1**	2	-1	**3**	4	-1							
***Anas platyrhynchos*** Mallard	1	0	**1**	1	0	0	1	0	**1**	1	0	0	0	1	NA	**1**	1	0	**3**	3	0							

“TOTAL MAT” = sum of relationships for material services; “TOTAL NON-MAT” = sum of relationships for non-material services; “TOTAL REG” = sum of relationships for regulating/maintenance services. Empty cells indicate no information found in the literature for relying on species with these NCPs. Potential crop* = potential crop (genetic resources); Wild use* = wild use medicinal, dye, fur; Mellifera* = all products from domestic bees; Linked to* = linked to endangered habitat; Dec* = decontamination; Reduce runoff* = reduce runoff from agroecosystems; Reduction of species damage* = Reduction of species damage and/or disease vector species. The full version of the table is available in the Appendix S3 (see 'Data availability'). Bold numbers indicate the cumulated score by category and with all NCPs.

**Figure 4:**
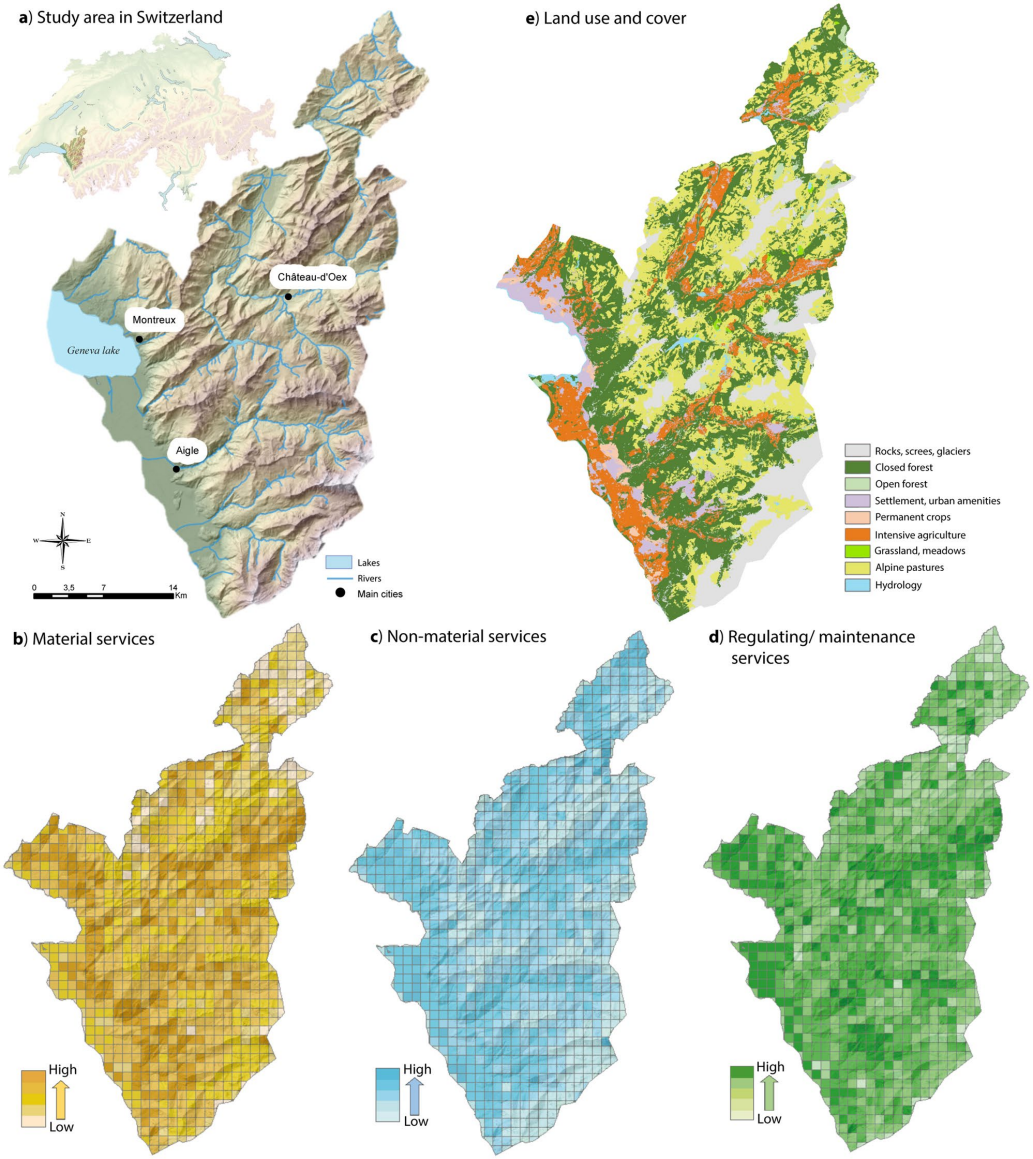
Example of an application of the species-NCPs relationship table for mapping NCP indices based on the relationships between the species (250 vertebrates and 1816 tracheophytes) and three NCP categories. (**a**) Location and topography of the study area (Box 1). (**b**), (**c**) and (**d**) Mapped NCP index at 1 km^2^ resolution for the three categories “material services”, “non-material services”, and “maintenance/regulating services”. Details on NCP index calculation are provided in Box 1. (**e**) Land use and land cover (LULC) map of the study area adapted from open access data of Giuliani et al.^76^.

Box 1: Example of the application of the species-NCPs relationship table to the spatial mapping of a global NCP index To illustrate one potential use of the species-NCPs relationship table (Appendix S3, see ‘Data availability’), we predicted the spatial distribution of each NCP based on the distributions of associated species from the table. We further combined individual NCP prediction maps into three NCP index maps, one per NCP category at the pixel level (1 km^2^ resolution) across a study area of the western Swiss Alps, where all species used had at least 10 occurrences during the last 50 years. This study area has been intensively studied^35^ and is characterized by a wide elevational and topographic gradients, heterogeneous land covers (Fig. 4a,e), resulting in a great species richness^36^. Based on occurrence data of vertebrate and tracheophyte species, we calculated an index displaying the potential for each NCP categories: material, non-material, regulating/maintenance services. More specifically for each 1 km^2^ pixel we: (1) summed the NCP score stored for each species and each category, (2) divided by the number of species observed, and (3) multiplied by the surface area of the pixel (border pixels were smaller than 1 km^2^ and needed to be adjusted). This index is only one illustration out of many possible indices and in this case allows comparing the representativeness of each NCP category according to the surface area and the number of species in each analytical unit (Fig. 4b–d).

Our results revealed different distribution patterns for the three categories of services across elevations, with material services rather prevailing at mid elevations (Fig. 4b), non-material services at low to mid elevations (Fig. 4c) and regulating and maintenance services showed some specific areas, spread across all elevations, with higher NCP index values (Fig. 4d). An explanation for the latter pattern could be the dominating occurrence of many common alluvial species (i.e. *Salix spp.*, *Populus spp.*) that have positive relationships with this regulating and maintenance NCP category, near rivers or lakes. The visual analysis of the NCP maps did not suggest any strong correlation with the patterns of land use and land cover (LULC; Fig. 4e), although high NCP values for material services were often found in closed forests and intensive agriculture areas, and high NCP values for regulating and maintenance services were observed in urban amenities and intensive agriculture areas. We noticed heterogeneous values of the NCP index inside a same LULC class, as it is the case for the regulating and maintenance services and the “rocks, screes, glaciers” LULC class. However, it is important to keep in mind that these example maps likely hold uncertainties due to the quality of species occurrences data obtained from different citizen-science databases, which are known to be heterogeneous and affected by sampling bias^37,38^

## Discussion

We presented the first large and exhaustive table of species-NCP relationships for vertebrates and tracheophytes in a whole region compiled from scientific literature, expert knowledge, and existing data. We further illustrated the potential importance and pivotal role of this tool for further analyses, such as spatial predictions of NCPs from individual species distributions or predictions across potentially large territories.

The proposed table illustrates the relationships currently documented in the scientific literature and considered by experts; thus, it does not represent all actual relationships and is expected to be continuously improved as these relationships are further documented. We presented a simple example illustrating the potential use of such a table to assess spatial variations in NCPs applied under current environmental conditions, which could equally be applied under future scenarios. The same assessment could also be based on species distribution model predictions^39^, and could account for variations in species’ abundances, as some species can cover large areas and occur under relatively high abundance, yet represent only a small fraction of species richness (typically dominant forest tree species). Different weights could also be assigned to the different species in future versions of the relationship table (e.g. level of toxicity for the wild food NCP), as these could prove useful in spatial conservation planning^17,30^. Furthermore, this analysis could be conducted at the level of individual NCPs for individual species, or at the level of species traits^25^ or species functions or roles in ecosystems^40^, depending on the study aims and the various interests of final users (e.g. stakeholders and decision-makers^41^).

Although the table represents a first version that will still benefit several improvements, it already shows that angiosperm species deliver numerous key services, such as being potential crops. Notably, the “wild food” NCPs had more negative than positive relationships (Fig. 1a), translating the greater proportion of species known to be non-edible or toxic in a regional flora (i.e. 214 negative vs 166 positive relationships for angiosperm groups), without necessarily reflecting the actual number of edible species (all species mentioned “toxic”, strongly or weakly took negative value). As fixed organisms, plants need a strong defence against herbivores, owing to their use of numerous toxic compounds (alkaloids, terpenoids, polyphenols)^42,43^. This raises the question whether their scientific interests mirror a popular interest^32^. From our results, the answer is currently no: although the number of “iconic species” and “species of scientific interest” NCPs were similar for the angiosperm group (23.4% and 24.3%, respectively); only 8.5% of angiosperm species were considered to have an interest for both scientists and the public.

Within the vertebrate groups, although most reptiles and amphibians (i.e. herptiles) are potentially edible, their protected status and the fact that they are not considered traditional food in many European countries (with exceptions, e.g. France) resulted in them not being considered to have a positive relationship with the “wild food” NCP. From the available literature, these two herptile groups only had relationships with the non-material NCPs. However, they could potentially play an increasing role in the near future to support the fight against insect-borne diseases^44^ (e.g. arboviruses such as chikungunya, dengue, or zika), which tend to shift their distribution towards higher latitudes under global warming^45^. Regarding vertebrates as a whole, the close to balanced positive and negative relationships observed for the “reduction of species damage and disease vector species” NCP (16 positive vs 15 negative for mammals, and 17 positive vs 14 negative for birds) can be explained by the fact that numerous species, including rodents or passerine birds, are considered to be species damaging cultures or affecting habitat structure^46–48^. Next, according to Horsley et al.^49^, “the term ‘iconic species’ is rarely explicitly defined but is generally used to describe species that share taxonomic similarities with the charismatic megafauna of flagship species”, and the “popular interest in vertebrates does not reflect extinction risk and is associated with bias in conservation investment”^33^. Therefore, we decided to avoid this bias by integrating all the species of interest for Switzerland population, according to the results from the Google search engine. Thus, common species such as the common raven (*Corvus* corax), red deer (*Cervus elaphus*), or Eurasian lynx (*Lynx lynx*) were considered as iconic species.

Although building such a relationship table was a first important step toward predicting NCPs from species, attributing a value (+ 1, 0, − 1, else NA) to every relationship can prove difficult. For example, attributing a negative relationship to the grey wolf (*Canis lupus*) with the “reduction of species damage and or disease vector species” NCP (Table 1B) can be seen as extremely controversial. For instance, as a “large predator”, the grey wolf can regulate a species with potential vector disease or inducing a land use perturbation (e.g. Elk (*Alces americanus*) in the Yellowstone Valley^50^). However, in Switzerland, the dominant view among national experts considered economic damages (attack of sheep herds) as more important, justifying the negative value (− 1) used in our study. A more neutral value could be considered in future studies. However, if attributing only one of three possible information values (+ 1, 0, − 1; else NA) was a useful first step, it may not sufficiently reflect the nature (e.g. intensity) of the relationship. This can be illustrated for the sparrow (*Passer* genus) and the wild boar (*Sus scrofa*), both of which had a negative relationship with the “reduction of species damage and disease vector species” NCP, but with fairly different disturbance effects (i.e. feeding on seeds before yield vs tilling soils in grasslands and maize plantations for feeding, respectively; expert pers. comm.). Such a table can only reflect our current knowledge, and therefore, must be used and interpreted with caution, also acknowledging the remaining unknowns and uncertainties. Future developments could attempt at using a real number instead of − 1, 0 or + 1 to fill the table, to translate the intensity of positive or negative contributions of different species to a same NCP, for instance to better account also for the different roles and importance of species in ecosystem functioning^51^, provided that sufficient information can be found in the literature.

## Conclusions

This study is the first to propose a comprehensive species-NCP relationship table, mapping the relationships between thousands of species and several key terrestrial NCPs. More importantly, it is the first study considering different types of relationships (positive, neutral or negative) between species and NCPs, allowing to complement the human-centred economic valuation of NCPs. This also avoids the risk to relate human well-being only to monetary values (i.e. green capitalism)^52^ and, instead , to also consider the intrinsic values of species^53^. Our results thus open interesting perspectives to better integrate human well-being with biodiversity in the context of the recent IPBES and Intergovernmental Panel on Climate Change (IPCC) reports^54,55^.

Furthermore, our study and the resulting table represent the first, yet crucial step towards predicting NCPs from species in space and time. Here, the focus was on two important taxonomic groups and their related terrestrial NCPs distributed across the Western Swiss Alps. Notably, each organismal group could have a separate methodology depending on the NCP considered. The methodology is applicable elsewhere; however, new references would be needed for new regions. Our specific case study (Box 1) illustrated the great advantages that such a table can provide as a key tool to map NCPs based on species distributions, such as being obtained from spatially-explicit surveys or species distribution model (SDM^56^) predictions. Thus, this approach bears great potential in predicting the future spatial distribution of NCPs, and therefore, in combining them with species range shifts in global change studies^15,17^. However, the sole use of NCP maps for conservation planning is not sufficient and should always be supported by in depth additional evaluations of the ecosystem functions, community characteristics and species protection status.

### Perspectives

If robustly quantified, these direct relationships between biodiversity (species) and NCPs, and associated spatial predictions, have the potential to become a cornerstone of conservation planning^57^ and more generally, of sustainable development^58^.

The proposed table should therefore be considered a first product (for now, focused on the Western Swiss Alps), which will highly benefit from further improvements as well as taxonomic and geographic expansions. For example, we did not find sufficient information to fill all cells of the relationship table for a few less known taxonomic subgroups (e.g. lycopodiophytes), for which it would be valuable to refine the study. It would also be important to expand it to other taxonomic groups (e.g. arthropods, gastropods, crustaceans, molluscs, fungi, lichens, bryophytes, etc.). Furthermore, although invasive alien species were not included here, it could be interesting to study their relationships with NCPs that could also be beneficial or detrimental for human wellbeing^59^, such as the black locust (*Robinia pseudoacacia*) that can harm natural ecosystems^60^ but also has positive attributes, such as soil stabilization, nitrogen fixation, consumable plants for humans and herds, or rotproof wood^61^.

Further developments could also integrate other NCPs, such as the scenic beauty^62^ or relations to soil nutrients (nitrogen, phosphorus) or carbon content^63,64^. Similarly, aquatic NCPs could also be considered (i.e. oxygen supplier^65^); however, insufficient information was found to complement the table for these (Appendix S2, see ‘Data availability’). The CICES classification^11^ would permit to reproduce our methodology for any sufficiently documented species-NCP system in the world.

Lastly, it would be interesting to relate not only single species with single NCPs, but also relate species assemblages with bundles of NCPs, as a way to better understand species-NCP interactions^66^. The next step could thus be to identify key NCP bundles^66^ and assess their link to species assemblages and their functions, still in the perspective of predicting NCPs spatially^23^. This approach might allow avoiding the focus on keystone or characteristic species only to identify potential key communities^40^, by instead predicting NCPs based on spatial aggregation of many species’ functionalities (as e.g. done to predict plant communities from species traits^67^). All these developments have the potential to contribute to the establishment of improved spatial conservation prioritization networks.

## Materials and methods

### Study area

The study area is a well-documented region of the Western Swiss Alps^35^ representative of the typical land cover types and topographic variation found in Western Switzerland (Fig. 4a). It spans an altitudinal range in the range of 372–3206 m and covers an area of 946 km^2^.

### Species selection

The species list encompasses the large majority of species occurring in the target study area (see filtering selection below) and is based on the network of Swiss national species information centers (www.infospecies.ch), compiling several scientific inventories, natural history collections, and citizen science data. In particular, several exhaustive species inventories (e.g. for plants, mammals, bats, several insects groups, bacteria, fungi and protists) were conducted across the study area over the last 20 years following random-stratified designs^35^. Among this first species list, we retained all tracheophyte (i.e*.* vascular plants) and vertebrate species with at least 10 occurrences in Switzerland between 1970 and 2020^68^. As the aim was to predict native NCPs, we excluded all alien species. The final species list included 1816 tracheophyte and 250 vertebrate species (Appendix S3, see ‘Data availability’). Closely-related tracheophyte species that proved difficult to differentiate were aggregated to the smallest common taxon, based on taxonomic expertise, to avoid mistakes due to misidentifications. No such corrections were necessary for vertebrates.

### NCPs selection

NCPs potentially linkable to each of the 2066 target species were selected both from the latest version of the European classification of ecosystem services (CICES V.5.1^11^) and from the Swiss classification of ecosystem services^69^. With the goal to initiate work on relating species and NCPs at the national scale, we decided to combine both EU and Swiss classifications to simplify results for national and global stakeholders (see Supplementary Table S2 for the correspondence between CICES and the Swiss classification^69^). As we were only interested in biotic NCPs with clear links to ecosystems, we removed all abiotic NCPs (e.g. solar energy; maintenance and regulation by inorganic natural chemical and physical processes; natural, abiotic characteristics of nature that enable intellectual interactions) and NCPs limiting nuisances of anthropogenic origin (e.g. smell reduction; noise attenuation). We also removed NCPs considering only marine and/or lacustrine species (e.g. plants cultivated by *in-situ* aquaculture as an energy source; regulation of the chemical condition of salt waters by living processes). The remaining biotic NCPs were then sorted by their feasibility according to the species groups studied. The table of the CICES NCPs used in this study can be found in the Appendix S2 (see ‘Data availability’).

We ended up with a list of 16 NCPs for tracheophyte and 9 for vertebrate species (Tables 2, 3). Selected NCPs were those having a relationship with at least one species of the corresponding taxonomic group (expert knowledge) following the CICES system^11^ and its 84 NCP classes (e.g. cultivated terrestrial plants for nutritional purposes, control of erosion rates, characteristics of living systems that are resonant for culture or heritage, etc.), which were further allocated to one of three categories (i.e. material, non-material, maintenance/regulating) (Table 2).

**Table 2:** Summary characteristics of the 17 Nature Contributions to People (NCPs; encompassing ‘ecosystem services’) considered in this article and related to the 1816 tracheophyte and 250 vertebrate species (16 NCPs for tracheophytes and 9 for vertebrates) in the Western Swiss Alps.

Category	NCP	Example goods and benefits	T	V	Sources
Material NCPs	Potential crop (genetic resource)	Crop wild relatives (CWR) are defined as those wild species that are closely related to crops and exchange genes with the latter. Therefore, CWR are an important part of the gene pool of crops. They also have a high potential for crop breeding as well as for their potential use as novel crops	X		R, E
	Solid wood	Processed wood (e.g. basketry, wooden tools, construction,woodworking, marquetry, stationary, …)	X		R, E
	Burned wood	Plants (including fungi, algae) grown as a source of energy	X		R, E
	Wild food	Wild plants, animals (terrestrial, aquatic, including fungi, algae) used for nutrition. Included toxic plants	X	X	R, E
	Wild use medicinal, dye, fur	Fibres and other materials from wild plants for direct use or processing (excluding genetic materials) like essential oils, macerate	X	X	R, E
	Mellifera use	Wild plants (terrestrial) used for nutrition of domestic bees or livestock	X		R, E, A
	Forage/pasture		X		R, E, A
Regulating/maintenance NCPs	Decontamination	Decontamination of polluted soil (i.e. Al, Ag, As, Be, Cr, Cu, Mn, Hg, Mo, Ni, Pb, Pd, Pt, Se, Zn, Naphtalène, radionuclides, hydrocarbons, pesticides and organic solvents)	X		R, E
	Riverbank erosion	Control of erosion rates	X	X	R, E
	Hedge for crop yield	Natural protection to improve the crop yield	X		R, E, A
	Reduction of species damage and disease vector species	Reduction of potential pest damage to planted fruits or vegetables. Regulation of species vectors of disease		X	R, E
	Reduce runoff from agroecosystems	Reduced damage costs nutrient runoff from agroecosystems	X		R, E, A
	Keystone species	Essential for other organisms and whose disappearance can lead to the disappearance of other species and greatly modify the functioning of ecosystems	X	X	R, E
	Reduce landslide	Reduced damage costs caused by avalanche (incl. wet snow slide), landslide (incl. Rock, slope erosion and ice fall), mudflow	X	X	R, E
Non-material NCPs	Scientific interest	Characteristics of living systems that enable scientific investigation or the creation of environmental and nature knowledge	X	X	R, A
	Iconic species	Elements of living systems that have symbolic meaning	X	X	R, A
	Linked to an endangered habitat	Characteristics or features of living systems that have an option or bequest value	X	X	R, E

NCP classification was based on the CICES (V.5.1)^11^ and on the ES Swiss classification^69^. In the “T” (Tracheophytes) and “V” (Vertebrates) columns, a cross indicates if the organismal group is linked, or not, to the NCP. The “Sources” column indicates the source used to document species-NCP relationship for each NCP: R = references, E = expert knowledge, A = comparison between species with literature and Google search engine results.

**Table 3:** NCP-specific methods used to attribute a positive, negative or neutral relationship of a species with the NCP, with distinction for plant and vertebrate species. (The full list of references is available in Supplementary Table S3).

Category	NCP	Methods
Material NCPs	Potential crop (genetic resource)	**Plants:** based on the CWR list in Switzerland (Boserup et al., 2021). *If a species was present in the CWR list, the species obtained a positive link (*+ *1), if else a neutral link (0)* | **Vertebrates:** NA
	Solid wood	**Plants:** based on expertise (wood exploited by "La Forestiere") and on bibliography (Dumé et al., 2018; Rameau et al., 1993). *If a species was cited for wood production, the species obtained a positive link (*+ *1), else a neutral link (0)* | **Vertebrates:** NA
	Burned wood	**Plants:** based on expertise (wood exploited by "La Forestiere") and on bibliography (Dumé et al., 2018; Rameau et al., 1993). *If a species was cited for firewood, the species obtained a positive link (*+ *1), else a neutral link (0)* | **Vertebrates:** NA
	Wild food	**Plants:** based on bibliography (Dumé et al., 2018; Günthardt et al., 2018; Rameau et al., 1993). *If a species was cited edible, the species obtained a positive link (*+ *1), if a species was cited toxic (with no parts of plant edible), the species obtained a negative link (− 1), if no specific information on the plant, the species obtained a neutral link(0)* | **Vertebrates:** based on federal law text and on expert statistics (Fauna expert—wildlife warden Simon Meier of Vaud canton). *If a species was cited edible or huntable for its caloric intake, the species obtained a positive link (*+ *1) with the NCP, else a neutral link (0)*
	Wild use medicinal, dye, fur	**Plants:** based on bibliography (see the full list of references in the Supplementary Table S3 | mainly on Dumé et al., 2018; Dal Cero et al., 2014; Rameau et al., 1993). *If a species was considered useful for medicine or dye in the bibliography, the species obtained a positive link (*+ *1), else a neutral link (0)* | **Vertebrates:** based on fauna (and statistics) expert at national level (warden Simon Meier of Vaud canton). *If a species was cited huntable for its fur or like trophy, the species obtained a positive link (*+ *1) with the NCP, else a neutral link (0)*
	Mellifera use	**Plants:** based on bibliography (see the list of references in the content “Bibliography”). *If a species was cited "mellifera plant" and useful for domestic bees, the species obtained a positive link (*+ *1), else a neutral link (0)* | **Vertebrates:** NA
	Forage/pasture	**Plants:** based on bibliography (see the full list of references in the Supplementary Table S3) and on TypoCH classes of Info Flora (*4.2. Thermophilic dry grasslands; 4.3.5. Acidic rough grazing; 4.5. Oily grasslands*) with dominant species and often influencing the physiognomy & characteristic species (furnished by Info Flora). *If a species was cited to be useful for the forage and for the nutrition of livestock in the bibliography and was present in the Typo-CH classes, the species obtained a positive link (*+ *1). If a species was cited as non-palatable or non-edible for livestock, the species obtained a negative link (-1), else a neutral link (0)* | **Vertebrates:** NA
Regulating/maintenance NCPs	Decontamination	**Plants:** based on bibliography (see the full list of references in the Supplementary Table S3) and on an exhaustive review of decontaminant plant (consulted: 26.05.2021). *If a species was considered as decontaminant, the species obtained a positive link (*+ *1), else a neutral link (0)* | **Vertebrates:** NA
	Riverbank erosion	**Plants:** based on bibliography (see the full list of references in the Supplementary Table S3) and on external expert review (Dr. Raymond Delarze—BEB study office). *If a species was considered as reducing riverbank erosion, the species obtained a positive link (*+ *1), else a neutral link (0)* | **Vertebrates:** based on external expert review (Dr. Raymond Delarze—BEB study office). *If a species was considered as reducing riverbank erosion risk, the species obtained a positive link (*+ *1). If a species increased the riverbank erosion risk, the species obtained a negative link (− 1), else a neutral link (0)*
	Hedge for crop yield	**Plants:** based on the TypoCH classes of Info Flora (*5.3. Bushy formations (mantle, thickets, hedges*)) with dominant species and often influencing the physiognomy and characteristic species and on external expert review (Dr. Raymond Delarze—BEB study office). *If a species was cited as improving crop growth, validated by the external expertise, and was present in the Typo-CH class, the species obtained a positive link (*+ *1), else a neutral link (0)* | **Vertebrates:** NA
	Reduction of species damage and disease vector species	**Plants:** NA|**Vertebrates:** based on the WSL webpage containing a list of pest species (consulted: 09.04.2021), on bibliography (see the full list of references in the Supplementary Table S3) and on expert statistics (species hunting for regulation | https://www.jagdstatistik.ch/; Data furnished by the wildlife warden of Vaud canton—Simon Meier). *If a species was considered as useful to limit the dispersal of disease or damage, the species obtained a positive link (*+ *1). If a species increased the damage or the contamination, the species obtained a negative link (− 1), else a neutral link (0). NB: few species could be regulated by hunt but could be equally useful for the regulation of damage species (e.g. Red fox (Vulpes vulpes) considered as regulator), in this case, the species obtained a positive link (*+ *1) with the NCP*
	Reduce runoff from agroecosystems	**Plants:** based on bibliography (see the full list of references in the Supplementary Table S3) and on the TypoCH classes of Info Flora (selecting species composing hedges—*5.3. Bushy formations (mantle, thickets, hedges)*) with dominant species and often influencing the physiognomy and characteristic species and on the external expert review (Dr. Raymond Delarze—BEB study office). *If a species was cited in the bibliography to reduce the runoff from agroecosystem, validated by the external expertise, and was present in the Typo-CH class, the species obtained a positive link (*+ *1), else a neutral link (0)* |**Vertebrates:** NA
	Keystone species	**Plants:** We considered a species like "keystone species” if the species was dominant in its environment and characteristic of its environment (based on the list of Delarze et al. 2015—"Dominant species and often influencing the physiognomy" + "characteristic species of the ecosystem"). *If a species was validated by the external expertise, the species obtained a positive link (*+ *1), else a neutral link (0)* |**Vertebrates:** based on external expert review (Dr. Raymond Delarze—BEB study office). *If a species was validated by the external expertise, the species obtained a positive link (*+ *1) with the NCP, else a neutral link (0)*
	Reduce landslide	**Plants:** based on bibliography (see the full list of references in the Supplementary Table S3), on expert statistics (SilvaProtect info) and on the external expert review (Dr. Raymond Delarze—BEB study office). *If a species was cited in the bibliography to reduce the landslide, avalanches validated by the external expertise, the species obtained a positive link (*+ *1), else a neutral link (0)* | **Vertebrates:** based on the WSL webpage containing a list of pest species (consulted: 09.04.2021), on the bibliography (see the full list of references in the Supplementary Table S3), and on the external expert review (Dr. Raymond Delarze—BEB study office). *If a species cited in the bibliography to reduce the landslide was validated by the external expertise, the species obtained a positive link (*+ *1). If species cited in the bibliography was listed into the WSL list like pest species and could increase the landslide and if it was validated by the external expertise, the species obtained a negative link (− 1) with the NCP, else a neutral link (0)*
Non-material NCPs	Scientific interest	**Plants and Vertebrates:** Develop a R script (available on github: https://github.com/PLREY/BD-x-NCP-relationship) to quantify the number of publications for each species based on Web of science (specialist website to store publications metadata) using ‘rwos’ package. Each branch (tracheophyte and vertebrate) was treated separately. We searched next query: Query < -((TS = ("scientific name" SAME species) OR TI = ("scientific name") OR AK = ("scientific name") AND PY = (1970–2020)) AND (DT = (Article OR Book OR Book Chapter)))"). The query (that can be submitted directly species by species with the website web of science) allowed to search the scientific name of each species into an article, book or book chapter stored between 1970–2020 range into web of science. *After to have separated species by taxonomic group, we ranked species by the number of times they were associated or mentioned to a publication. Only the top quartile species (i.e. higher than the 3rd quartile) obtained a positive link (*+ *1), else a neutral link (0)*
	Iconic species	**Plants and Vertebrates:** Develop a R script (available on github: https://github.com/PLREY/BD-x-NCP-relationship) to quantify the number of results reflecting which species are considered like "cultural icon", "emblematic species", determining species of interest for Switzerland population. This R analytical approach was based on Google analytics, more precisely on the count of each link (French; Italian; German; English language with a ".ch" domain) containing all following terms: "scientific name"; "emblematic", "Switzerland" and "nature". Each branch (tracheophyte and vertebrate) was treated separately. *After separating species by taxonomic group, we ranked species by the number of associated weblinks, species with value higher than the 3rd quartile obtained a positive link (*+ *1), else a neutral link (0)*
	Linked to an endangered habitat	**Plants and Vertebrates:** based on Emerald species list defined like “species with intrinsic value that have the potential to contribute to the maintenance or recovery of species and habitats at a favorable conservation status” (consulted: 20.05.2021). *If a species was indicated "Emerald", the species obtained a positive link (*+ *1), else a neutral link (0)*

### Filling the table of species-NCP relationships

The species-NCP relationships’ table was filled by assigning a value (positive, neutral, or negative) to each cell, and NA when no information could be found or if a species was not concerned by an NCP (Table 3). To obtain the final table, three complementary strategies were combined to retrieve information from heterogeneous sources: (1) a screening of the scientific literature, (2) an expert-knowledge assessment, and (3) a comparison between species with literature and Google search engine results when possible (Tables 2, 3). A specific ‘third quartile’ methodology was used to assign values to the “Scientific interest” and “Iconic species” NCPs. Here, based on the cumulative distribution of the number of references identified by the google/web of science search engines, we used the third quartile threshold to identify the species that were the most often referenced. Assigning a negative value was not possible for the NCPs “solid wood”, “burned wood”, “mellifera (for domestic species)”, “decontamination”, “hedge for crop yield”, and all non-material services.

The final table contains the following columns: scientific name, vernacular names (English, French, German and Italian), class, order, family, organismal group (French), organismal group (English) (i.e. branch), IUCN threat status in Switzerland^70–75^, number of occurrences in Switzerland between 1970 and 2020, the value for each NCP (+ 1/0/ − 1/NA), the total value for each NCP category (e.g. material, non-material, and regulating/maintenance services), the total value across species for all NCPs considered, the number of positive and negative relationships for each category, and all NCPs considered (Table 1; full relationship table between species and NCPs available on the Appendix S3; see ‘Data availability’).

### Species-NCP relationship analyses

We graphically summarized the species-NCP relationships by using a positive/negative bar plot. A sample of the relationship table with the top-scored species is provided to visualize the content of the table. Finally, to illustrate a potential simple application of the relationship table to predict NCPs spatially, we provided a case-study box focusing on the spatial predictions (at a pixel resolution of 1 km^2^) of the three main NCPs categories (material, non-material, regulating/maintenance) based on observed species occurrence data within the Swiss Western Alps (Box 1).

All figures were designed by authors using R (v.4.0.5) and Arcgis (v.10.8.1) software and improved with Adobe Illustrator (v.27.3.1). Figure 4e was adapted from open access data of Giuliani et al.^76^.

## Supplementary Information

Supplementary Information.

## Data Availability

Data from the Infospecies database can be publicly consulted from gbif repository at https://doi.org/10.15468/mzzz8z. Appendix S1: Correspondence and aggregation for tracheophytes names (10.6084/m9.figshare.19362029). Appendix S2: Detailed information to establish relationship methodology between biodiversity and NCPs (10.6084/m9.figshare.19222830). Appendix S3: Biodiversity and NCP relationship table (10.6084/m9.figshare.19182632).

## Code availability

Appendix S4: Biodiversity x NCPs relationship—script R (10.6084/m9.figshare.19362011).
