# Supplementary material for: Linking plant and vertebrate species to Nature’s Contributions to People in the Swiss Alps
Source: Sci Rep. 2023 May 5;13:7312. doi: 10.1038/s41598-023-34236-2 (PMC10163046; doi:10.1038/s41598-023-34236-2)
Supplement: Supplementary file 1 — Supplementary Information. [file 41598_2023_34236_MOESM1_ESM.docx]

# Supplementary information

Manuscript title: Linking plant and vertebrate species to Nature’s Contributions to People in the Swiss Alps

Authors: [hidden for peer-review]

Journal: Scientific reports

Table S1: Aggregation table of the 65 land use and land cover classes into 8 main categories (color blocks in the table). Data from Giuliani et al. (2022).

Giuliani, G., Rodila, D., Külling, N., Maggini, R., & Lehmann, A. (2022). Downscaling Switzerland land use/land cover data using nearest neighbors and an expert system. Land, 11(5), 615.

Table S2: Correspondence between CICES (v.5.1) and FOEN (Staub et al. 2011) to work on NCP methodology.

BAFU, 2011. Indicators for Ecosystem Goods and Services: Systematic, Methodology and Recommendations for Environmental Information Related to Well-Being (in original language (French): « Indicateurs pour les biens et services écosystémiques : Systématique, méthodologie et recommandations relatives aux informations sur l’environnement liées au bien-être »). <http://www.environnement-suisse.ch/uw-1102-f>

CICES (Common International Classification of Ecosystem Services), 2018. Guidance & Spreadsheet v.5.1. <https://cices.eu/resources/>

Table S3: List of references used to attribute relationship value between species and NCP.

| **NCP-ID** | **References** |
| --- | --- |
| **Potential crop (genetic ressources)** | Julie Boserup, Sibyl Rometsch, & Sylvain Aubry (2021). La conservation des Crop Wild Relatives (CWR). Infoflora & OFEV source |
| **Solid wood** | Dumé, G., Gauberville, C., Mansion, D., & Rameau J. C., (2018). *Flore forestière française: Plaines et collines* (No. 1). Forêt privée française. Nouvelle édition |
|  | Rameau, J. C., Mansion, D., & Dumé, G. (1993). *Flore forestière française: Montagnes* (No. 2). Forêt privée française. |
| **Burned wood** | Dumé, G., Gauberville, C., Mansion, D., & Rameau J. C., (2018). *Flore forestière française: Plaines et collines* (No. 1). Forêt privée française. Nouvelle édition |
|  | Rameau, J. C., Mansion, D., & Dumé, G. (1993). *Flore forestière française: Montagnes* (No. 2). Forêt privée française. |
| **Wild food** | Dumé, G., Gauberville, C., Mansion, D., & Rameau J. C., (2018). *Flore forestière française: Plaines et collines* (No. 1). Forêt privée française. Nouvelle édition |
|  | Günthardt, B. F., Hollender, J., Hungerbühler, K., Scheringer, M., Bucheli., T. D. (2018). Comprehensive Toxic Plants–Phytotoxins Database and Its Application in Assessing Aquatic Micropollution Potential. J. Agric. Food Chem. doi/10.1021/acs.jafc.8b01639 |
|  | Meier, S. (22.04.2021). statistical center of Hunting - https://www.jagdstatistik.ch/fr/ [Base de données]. Office fédéral de l’environnement, Division Biodiversité et paysage, Section Faune sauvage et conservation des espèces. |
|  | Rameau, J. C., Mansion, D., & Dumé, G. (1993). *Flore forestière française: Montagnes* (No. 2). Forêt privée française. |
| **Wild use medicinal, dye, fur** | Dal Cero, M., Saller, R., & Weckerle, C. S. (2014). The use of the local flora in Switzerland: a comparison of past and recent medicinal plant knowledge. *Journal of ethnopharmacology*, *151*(1), 253-264. |
|  | Dumé, G., Gauberville, C., Mansion, D., & Rameau J. C., (2018). *Flore forestière française: Plaines et collines* (No. 1). Forêt privée française. Nouvelle édition |
|  | Rameau, J. C., Mansion, D., & Dumé, G. (1993). *Flore forestière française: Montagnes* (No. 2). Forêt privée française. |
| **Mellifera (for domestic species)** | Clément, H., Le Conte, Y., Le Barbançon, J. M., Vaissière, B., Bonnaffé, P., Reeb, C., Fert, G., Bruneau, E., Domerego, R., & Ratia, G., (2009). Le traité Rustica de l'apiculture, *Paris, Éditions Rustica*, févr. 2004, 528 p., ISBN 2-84038-421-3 |
|  | Desprets, A., Hemptinne, J., &  Hemptinne, J. L., (1985). Une gestion de l’environnement pour une apiculture florissante. *Ath, Association pour la promotion scientifique et pédagogique du département agriculture de l'institut provincial supérieur industriel du Hainaut, 74 p.* |
|  | Dumé, G., Gauberville, C., Mansion, D., & Rameau J. C., (2018). *Flore forestière française: Plaines et collines* (No. 1). Forêt privée française. Nouvelle édition |
|  | Hurpin, J., (1978), La flore mellifère de France : Étude des principales plantes mellifères et pollinifères des diverses régions de France, leur culture, leur valeur agricole et industrielle, leur importance comparée dans la production du miel. *Syndicat National d'Apiculture, 2e éd., 117 p.* |
|  | Rameau, J. C., Mansion, D., & Dumé, G. (1993). *Flore forestière française: Montagnes* (No. 2). Forêt privée française. |
| **Forage/pasture** | Delarze, R., Gonseth, Y., Eggenberg, S., & Vust, M. (2015). Guide des milieux naturels de Suisse: écologie, menaces, espèces caractéristiques. Lausanne, Switzerland: Rossolis. 440p. 3ème ed. |
|  | Dumé, G., Gauberville, C., Mansion, D., & Rameau J. C., (2018). *Flore forestière française: Plaines et collines* (No. 1). Forêt privée française. Nouvelle édition |
| **Decontamination** | Baker, A. J., & Brooks, R. (1989). Terrestrial higher plants which hyperaccumulate metallic elements. A review of their distribution, ecology and phytochemistry. Biorecovery., 1(2), 81-126. |
|  | Collins, C. D. (2007). Implementing phytoremediation of petroleum hydrocarbons. In Phytoremediation (pp. 99-108). Humana Press. |
|  | Delorme, T. A., Gagliardi, J. V., Angle, J. S., & Chaney, R. L. (2001). Influence of the zinc hyperaccumulator Thlaspi caerulescens J. & C. Presl. and the nonmetal accumulator Trifolium pratense L. on soil microbial populations. Canadian journal of microbiology, 47(8), 773-776. |
|  | Ernst, W. H. O. (1990). Mine vegetation in Europe. Heavy metal tolerance in plants: Evolutionary aspects, 18, 21-38. |
|  | Famulari, S., & Witz, K. (2015). A user-friendly phytoremediation database: creating the searchable database, the users, and the broader implications. International journal of phytoremediation, 17(8), 737-744. |
|  | Fiegl, J. L., McDonnell, B. P., Kostel, J. A., Finster, M. E., & Gray, K. (2010). A Resource Guide: The Phytoremediation of Lead in Urban, Residential Soils. |
|  | Hutchinson, S. L., Banks, M. K., & Schwab, A. P. (2001). Phytoremediation of aged petroleum sludge: effect of inorganic fertilizer. Journal of environmental quality, 30(2), 395-403. |
|  | Lamoureux, C. (2007). Design écologique pour le traitement des eaux usées dans les petites collectivités nordiques et isolées: le cas d'une communauté crie de la région de la Baie James. |
|  | Lombi, E., Zhao, F. J., Dunham, S. J., & McGrath, S. P. (2001). Phytoremediation of heavy metal–contaminated soils: Natural hyperaccumulation versus chemically enhanced phytoextraction. Journal of Environmental Quality, 30(6), 1919-1926. |
|  | McCutcheon, S.C. & Schnoor, J.L. (2003). Overview of phytotransformation and control of wastes. In Phytoremediation: Transformation and Control of Contaminants (ed J.L. Schnoor). John Wiley & Sons, Inc., Hoboken, New Jersey. |
|  | Negri, M. C., & Hinchman, R. R. (2000). The use of plants for the treatment of radionuclides (pp. 107-132). John Wiley & Sons, Inc., New York. |
|  | Prasad, M. N. V. (2005). Nickelophilous plants and their significance in phytotechnologies. Brazilian Journal of Plant Physiology, 17(1), 113-128. |
|  | Schmidt, U. (2003). Enhancing phytoextraction: the effect of chemical soil manipulation on mobility, plant accumulation, and leaching of heavy metals. Journal of Environmental Quality, 32(6), 1939-1954. |
|  | Siciliano, S. D., Germida, J. J., Banks, K., & Greer, C. W. (2003). Changes in microbial community composition and function during a polyaromatic hydrocarbon phytoremediation field trial. Applied and Environmental Microbiology, 69(1), 483-489. |
|  | Srivastav, R. K., Gupta, S. K., Nigam, K. D. P., & Vasudevan, P. (1994). Treatment of chromium and nickel in wastewater by using aquatic plants. Water Research, 28(7), 1631-1638. |
|  | Yu, X. Z., Zhou, P. H., & Yang, Y. M. (2006). The potential for phytoremediation of iron cyanide complex by willows. Ecotoxicology, 15(5), 461-467. |
| **Riverbank erosion** | Bariteau, L., Bouchard, D., Gagnon, G., Levasseur, M., Lapointe, S., & Bérubé, M. (2013). A riverbank erosion control method with environmental value. Ecological Engineering, 58, 384-392. https://doi.org/10.1016/j.ecoleng.2013.06.004 |
|  | Graf, C., Böll, A., Graf, F., & Dousse, M. (2003). Des plantes pour lutter contre l'érosion et les glissements en surface. Institut fédéral de recherches WSL. |
|  | Dumé, G., Gauberville, C., Mansion, D., & Rameau J. C., (2018). Flore forestière française: Plaines et collines (No. 1). Forêt privée française. Nouvelle édition |
|  | Rameau, J. C., Mansion, D., & Dumé, G. (1993). Flore forestière française: Montagnes (No. 2). Forêt privée française. |
| **Hedge for crop yield** | Altieri, M. A. (1999). The ecological role of biodiversity in agroecosystems. In Invertebrate biodiversity as bioindicators of sustainable landscapes (pp. 19-31). Elsevier. |
|  | Delarze, R., Gonseth, Y., Eggenberg, S., & Vust, M. (2015). Guide des milieux naturels de Suisse: écologie, menaces, espèces caractéristiques. Lausanne, Switzerland: Rossolis. 440p. 3ème ed. |
| **Reduction of species damage and disease vector species** | Charbonnier, Y., Papura, D., Touzot, O., Rhouy, N., Sentenac, G., & Rusch, A. (2021). Pest control services provided by bats in vineyard landscapes. Agriculture, Ecosystems & Environment, 306, 107207. |
|  | García, D., Miñarro, M., & Martínez-Sastre, R. (2018). Birds as suppliers of pest control in cider apple orchards: Avian biodiversity drivers and insectivory effect. Agriculture, ecosystems & environment, 254, 233-243. |
|  | Landolt, W., et al., (WSL). (2008). Diagnostic en ligne - WSL. waldschutz.wsl.ch. https://waldschutz.wsl.ch/fr/diagnostic-et-conseil/diagnostic-en-ligne.html |
|  | Peisley, R. K., Saunders, M. E., & Luck, G. W. (2015). A systematic review of the benefits and costs of bird and insect activity in agroecosystems. Springer Science Reviews, 3(2), 113-125. |
|  | Pejchar, L., Clough, Y., Ekroos, J., Nicholas, K. A., Olsson, O. L. A., Ram, D., ... & Smith, H. G. (2018). Net effects of birds in agroecosystems. BioScience, 68(11), 896-904. |
|  | Puig-Montserrat, X., Torre, I., López-Baucells, A., Guerrieri, E., Monti, M. M., Ràfols-García, R., ... & Flaquer, C. (2015). Pest control service provided by bats in Mediterranean rice paddies: linking agroecosystems structure to ecological functions. Mammalian Biology, 80(3), 237-245. |
|  | Riccucci, M., & Lanza, B. (2014). Bats and insect pest control: a review. Vespertilio, 17, 161-169. |
| **Reduce runoff from agroecosystems** | Altieri, M. A. (1999). The ecological role of biodiversity in agroecosystems. In Invertebrate biodiversity as bioindicators of sustainable landscapes (pp. 19-31). Elsevier. |
|  | Delarze, R., Gonseth, Y., Eggenberg, S., & Vust, M. (2015). Guide des milieux naturels de Suisse: écologie, menaces, espèces caractéristiques. Lausanne, Switzerland: Rossolis. 440p. 3ème ed. |
|  | Dumé, G., Gauberville, C., Mansion, D., & Rameau J. C., (2018). Flore forestière française: Plaines et collines (No. 1). Forêt privée française. Nouvelle édition |
| **Keystone species** | Delarze, R., Gonseth, Y., Eggenberg, S., & Vust, M. (2015). Guide des milieux naturels de Suisse: écologie, menaces, espèces caractéristiques. Lausanne, Switzerland: Rossolis. 440p. 3ème ed. |
| **Reduce landslide** | Graf, C., Böll, A., Graf, F., & Dousse, M. (2003). Des plantes pour lutter contre l'érosion et les glissements en surface. Institut fédéral de recherches WSL. |
|  | Dumé, G., Gauberville, C., Mansion, D., & Rameau J. C., (2018). Flore forestière française: Plaines et collines (No. 1). Forêt privée française. Nouvelle édition |
|  | Rameau, J. C., Mansion, D., & Dumé, G. (1993). Flore forestière française: Montagnes (No. 2). Forêt privée française. |
|  | Landoldt et al., 2008 |
| **Scientific interest** | NA |
| **Iconic species** | Affouard, A., Goëau, H., Bonnet, P., Lombardo, J. C., & Joly, A. (2017) Pl@ntnet app in the era of deep learning. In ICLR: International Conference on Learning Representations. |
|  | Joly, A., Bonnet, P., Goëau, H., Barbe, J., Selmi, S., Champ, J., Dufour-Kowalski, S., Affouard, A., Carré, J., Molino, J.F. and Boujemaa, N., 2016. A look inside the Pl@ntNet experience. Multimedia Systems, 22(6), pp.751-766. |
| **Linked with an endangered habitat** | OFEV 2019 : Liste des espèces et des milieux prioritaires au niveau national. Espèces et milieux prioritaires pour la conservation en Suisse. Office fédéral de l’environnement, Berne. L’environnement pratique n° 1709 : 98 p. |
